# Supplementary material for: Transcriptome Analysis Revealed the Key Genes and Pathways Involved in Seed Germination of Maize Tolerant to Deep-Sowing
Source: Plants (Basel). 2022 Jan 28;11(3):359. doi: 10.3390/plants11030359 (PMC8838884; doi:10.3390/plants11030359)
Supplement: Supplementary file 1 [file plants-11-00359-s001.zip › Table S7. Primer sequences of genes used for qRT-PCR detection.pdf]

Table S7. Primer sequences of genes used for qRT-PCR detection

Primers	Sequences (5’-3’)
***Zmβ-TUB*-F**	CTACCTCACGGCATCTGCTATGT
***Zmβ-TUB*-R**	GTCACACACACTCGACTTCACG
**GRMZM2G405459-F**	CTCCATCAAGTCCCAGGTG
**GRMZM2G405459-R**	TGCGGAAGTAGCCGTTGTC
**GRMZM2G030790-F**	AAAGTCCAAGAAGGCTAAAG
**GRMZM2G030790-R**	CGCCCGTCTGGTTGTAG
**GRMZM5G809195-F**	CCTCGTCGGTGGTGAAG
**GRMZM5G809195-R**	CATGATCCTCAGCTTCTTGC
**GRMZM2G078465-F**	CGTGCTCGTCAACTCGTTCTAC
**GRMZM2G078465-R**	CTTGCTTTCTGCCGCCATT
**GRMZM2G103055-F**	TCTGTTCCTCGTCCTTCTCG
**GRMZM2G103055-R**	CTTGCCCATCAGGAAGTTGTA
**GRMZM2G093286-F**	ACCACAGCACAAACGCAACG
**GRMZM2G093286-R**	CGAAGATGGCGAGGTAGGC
**GRMZM2G043338-F**	CGCTCACGGACTGGAAACTC
**GRMZM2G043338-R**	CAGTCTCGCCAGTAGCAAGGTC
**GRMZM2G067743-F**	TGTGACATTTTCGTTCAGGTTG
**GRMZM2G067743-R**	AAACGATCAAAATGACAGAGCC
**GRMZM2G337229-F**	GGTGAGATGCTACGGTTTC
**GRMZM2G337229-R**	GATTTGTCGCTTGGGTTTA
**GRMZM2G096435-F**	CCACAGCACAGCGAGCAT
**GRMZM2G096435-R**	CGTGAAGTAGCGGTACATCCAG
**GRMZM2G480954-F**	ACGGCATCCAGAGCAAGG
**GRMZM2G480954-R**	CATAACAGTCAACGAGCAAACC
**GRMZM2G168474-F**	GCTCAAGGGCAGCAAGCAG
**GRMZM2G168474-R**	TGGTGAACTCGGACAGGAACAT
