# Supplementary figures and images for: Transcriptome Analysis Revealed the Key Genes and Pathways Involved in Seed Germination of Maize Tolerant to Deep-Sowing
Source: Plants (Basel). 2022 Jan 28;11(3):359. doi: 10.3390/plants11030359 (PMC8838884; doi:10.3390/plants11030359)

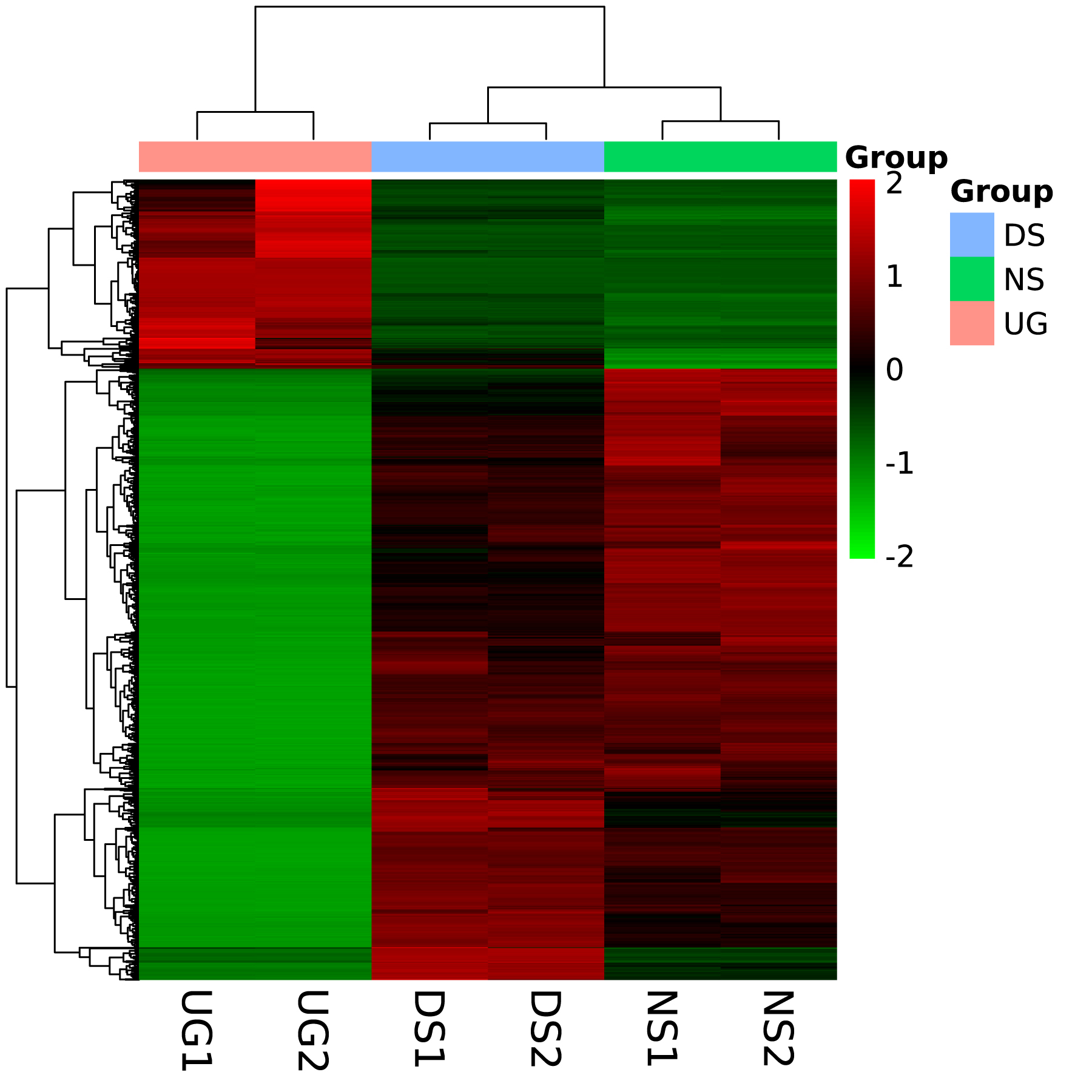

Supplement: Supplementary file 1 [file plants-11-00359-s001.zip › Figure S1.jpg]

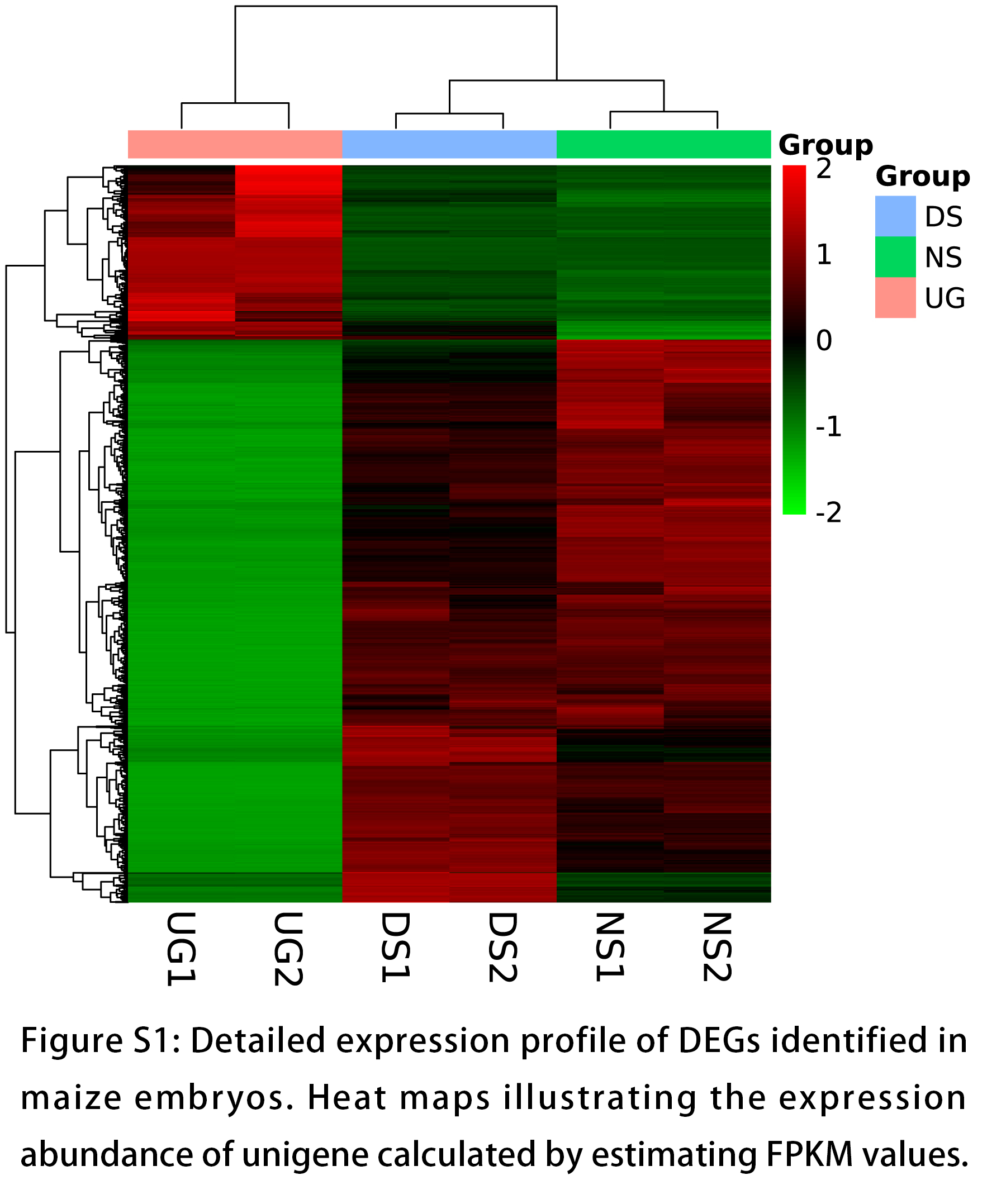

Supplement: Supplementary file 1 [file plants-11-00359-s001.zip › plants-1543925-resubmit-supplementary.png]
